# Investigation of anti-cancer mechanisms by comparative analysis of naked mole rat and rat

**DOI:** 10.1186/1752-0509-7-S2-S5

**Published:** 2013-10-14

**Authors:** Zhiyuan Yang, Yan Zhang, Luonan Chen

**Affiliations:** 1 Key Laboratory of Systems Biology, SIBS-Novo Nordisk Translational Research Centre for PreDiabetes, Shanghai Institutes for Biological Sciences, Chinese Academy of Sciences, Shanghai 200031, China; 2 Key Laboratory of Nutrition and Metabolism, Institute for Nutritional Sciences, Shanghai Institutes for Biological Sciences, Chinese Academy of Sciences, Shanghai 200031, China; 3 Collaborative Research Center for Innovative Mathematical Modelling, Institute of Industrial Science, The University of Tokyo, Tokyo 153-8505, Japan

## Abstract

**Background:**

The naked mole rats (NMRs) are small-sized underground rodents with plenty of unusual traits. Their life expectancy can be up to thirty years, more than seven times longer than laboratory rat. Furthermore, they are resistant to both congenital and experimentally induced cancer genesis. These peculiar physiological and pathological characteristics allow them to become a suitable model for cancer and aging research.

**Results:**

In this paper, we carried out a genome-wide comparative analysis of rat and NMR using the recently published genome sequence of NMR. First, we identified all the rat-NMR orthologous genes and specific genes within each of them. The expanded and contracted numbers of protein families in NMR were also analyzed when compared to rat. Seven cancer-related protein families appeared to be significantly expanded, whereas several receptor families were found to be contracted in NMR. We then chose those rat genes that were inexistent in NMR and adopted KEGG pathway database to investigate the metabolic processes in which their proteins may be involved. These genes were significantly enriched in two rat cancer pathways, "Pathway in cancer" and "Bladder cancer". In the rat "Pathway in cancer", 9 out of 14 paths leading to evading apoptosis appeared to be affected in NMR. In addition, a significant number of other NMR-missing genes enriched in several cancer-related pathways have been known to be related to a variety of cancers, implying that many of them may be also related to tumorigenesis in mammals. Finally, investigation of sequence variations among orthologous proteins between rat and NMR revealed that significant fragment insertions/deletions within important functional domains were present in some NMR proteins, which might lead to expressional and/or functional changes of these genes in different species.

**Conclusions:**

Overall, this study provides insights into understanding the possible anti-cancer mechanisms of NMR as well as searching for new cancer-related candidate genes.

## Background

The naked mole rats (NMRs, *Heterocephalus glaber*) are mouse-sized subterranean rodents native to East Africa [1]. They have an exceptional set of physiological traits that make them adapt to living in the underground of droughty desert. They are becoming one of the most extraordinary organisms known to science [2].

NMRs are the longest-lived rodent known till now and their maximum lifespan can be up to thirty years [3]. By contrast, other similar-sized rodents such as mouse possess a life expectancy of only four years, which is far less than that of NMR. Previously published studies have indicated that the longevity of NMR was possibly because of the negligible decrement of age-related physiological characteristic along with their lifetime, such as declining fertility and mortality rate [4].

Besides delayed senescence, NMRs are remarkably resistant to both congenital and experimentally induced cancer genesis [5]. Cancer is a group of complex polygenic diseases that commonly affect lots of vertebrates, and is constantly considered to be an inevitable accompanied by senescence. Cancer is the second dominant cause of mortality in the world, which cause 7.6 million of death estimated by World Health Organization [6]. It has been recognized for quite a long time that cancer genesis is closely related to tumour suppressor genes and oncogenes [7]. Identifying the function of the added genes may bring us in another way to explore the regulatory network of the cancer process. In addition, the mechanisms of cancer resistance present in NMR are not thoroughly clear. Thus, identification of NMR genes closely implicated in cancer may provide us effective clues for delineating causes of cancer proneness and studying anti-cancer properties for mammalian organisms.

NMRs possess several other special physiological characteristics as well. Although NMRs belong to the order of rodentia, they are actually poikilothermal animals whose body temperatures vary continuously following the environment [8]. Furthermore, NMRs are insensitive to certain types of pain [9] and acid [10], and are well adapted to the underground surrounding at an extremely low oxygen concentration (10%-15%) [11].

Recently, using high-throughput next generation sequencing techniques, the genome of NMR has been sequenced. These excellent resources provide great opportunities for understanding the exceptional characteristics of NMR and improve biological and biomedical studies. Previously, some genes have been identified to be related to some of these unusual characteristics, e.g., the telomerase reverse transcriptase (TERT) gene and some other genes, which may be involved in extended longevity mechanisms of NMR [12]. However, investigation of the genomic information of NMR at a systems-biology level is still lacking, which may provide additional information to uncover the molecular mechanisms for the extraordinary traits (e.g., anti-cancer) of NMR.

In this paper, a comparative genomics study was carried out to explore the genes that were either common between rat and NMR, or specific to each of them. We divided these genes into three groups: common genes, genes only present in NMR and genes only present in rat. We then used the Pfam database to identify the events of gain or loss of different protein families between these two species. In addition, the Kyoto Encyclopedia of Genes and Genomes (KEGG) database was used to study the rat pathways in which the NMR-missing genes participate. A significant number of these genes were found to be enriched in the pathways related to the exceptional characteristics of NMR (such as cancer pathways), many of which have been previously reported to be associated with various cancers. Finally, we analyzed the sequence variations (such as domain insertion/deletion) of orthologous proteins to investigate the potentially expressional and/or functional alternations of them between rat and NMR. Overall, our data not only help unveil the cancer resistance mechanisms of NMR but provide insights into identifying new cancer-related genes.

## Methods

### Genome, database and resources

The complete set of annotated rat and NMR protein sequences were obtained from the UniProt database (http://www.uniprot.org/). For those genes with alternative splicing variants, proteins with the smallest PE value (which means the most possibility for the existence of the proteins) and the longest length were chosen to represent the gene-encoding protein sequences. A total of 20835 and 21553 proteins corresponding to their genes were finally obtained for rat and NMR, respectively.

The file containing the whole pathways of rat was downloaded from the KEGG database (http://www.genome.jp/kegg/pathway.html). The Online Mendelian Inheritance in Man (OMIM) database (http://www.ncbi.nlm.nih.gov/omim) was used to analyze the relationship between cancers and human orthologs of genes absent in NMR. Furthermore, the expression data of these genes in rat tissues were obtained from the Gene Expression Atlas (GXA) database (http://www.ebi.ac.uk/gxa/), which was used to identify whether or not the expression levels of these genes were related to cancer development.

### Identification of orthologous genes between rat and NMR

To analyze the orthologous gene pairs between rat and NMR, we employed the complete set of annotated proteins of one organism as queries to search for orthologs in the other species via BLASTP with a cut-off of E-value ≤ 1e-6. Orthologous genes were further defined as bidirectional best hits.

On the basis of identified orthologs between rat and NMR, we dissected all the genes into three classifications:

(1) Class I: the Shared genes, which were shared between rat and NMR;

(2) Class II: the NMR-missing genes, which were absent in NMR but present in rat;

(3) Class III: the NMR unique genes, which were found in NMR but missing in rat.

### Pfam analysis

Considering that the conserved domains in a protein could provide information for its function and evolutionary dynamics, we used the Pfam database [13] (http://pfam.sanger.ac.uk/search), which collected a large collection of protein families, to search for gain or loss events of different protein families between these two species.

All the proteins of rat and NMR were searched against Pfam database with a cut-off of E-value ≤ 1e-5. For each protein, if two or more Pfam families were available, only the one with the smallest E-value was selected. The number of each protein family in rat and NMR was then calculated respectively.

### KEGG pathway analysis of Class II genes

We further dissected the pathways containing Class II genes using the KEGG database resource, which is a collection of manually curated pathway maps according to current knowledge on protein-protein interactions [14].

First, each gene of Class II was mapped to their pathways. The p-value of the enrichment of NMR-missing genes in each pathway was then calculated by hypergeometric distribution test. Moreover, considering that KEGG pathways were composed of nodes which were actually modules including single gene or multiple functionally similar genes, we further analyzed the number and percentage of the nodes containing Class II genes in each of the enriched pathways.

### Sequence variation analysis of Class I genes

Although Class I genes were considered as orthologous genes between rat and NMR, sequence variations had been previously observed for certain proteins. For example, the glutathione peroxidase 1 (GPx1), which is highly expressed in mouse liver and kidney, has an early stop codon in NMR. Such a variation results in the lack of the C-terminal part and may be related to an order of magnitude lower activity in NMR tissues [15]. Thus, it would be useful to further study the orthologous genes between the two organisms for potential changes with regard to their function and/or regulation.

To systematically investigate such deletion or insertion events, we analyzed the BLASTP sequence alignment results for each Class I gene in NMR, and focused on gap-related parameters, such as alignment length, number of mismatches, and percentage of identical matches, to calculate the lengths of sequence insertions/deletions. Protein pairs of rat and NMR proteins were chosen at a cut-off of gap length>25 and percentage of mismatches <10%. To avoid incorrect protein annotation of NMR, the NMR proteins with significant fragment deletion were searched against the NMR genome using TBLASTN for further verification of the absence of these segments. Finally, all selected proteins were searched against the Conserved Domains Database (http://www.ncbi.nlm.nih.gov/Structure/cdd/cdd.shtml) to identify their functional domains.

## Results

### Distribution of orthologous genes in rat and NMR

**Figure 1:**
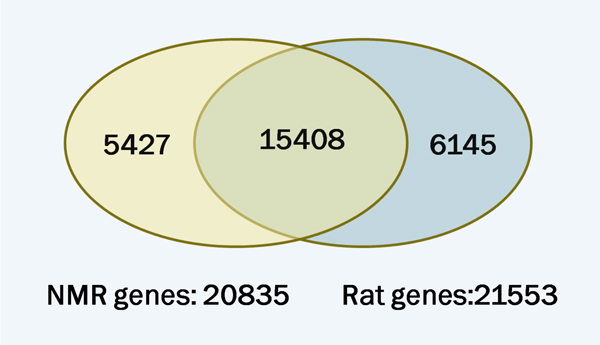
**Venn diagram of rat and NMR genes**. A total of 20835 NMR genes and 21553 rat genes were obtained. Among them, 15408 genes were detected in both species.

Figure 1 shows the Venn diagram between rat and NMR genes. A set of 15408 genes (Class I) were found in both rat and NMR, which occupied 73.9% of the total predicted NMR genes. On the other hand, 6145 rat genes (Class II) lacked orthologs in NMR. In contrast, 5427 genes (Class III) appeared to be unique in NMR. Nevertheless, considering that the number of NMR orthologous genes(Class I) is only about 71.5% of that of the rat total genes, it is possible that some genes may be mis-annotated in the currently released version of NMR genome.

### Pfam analysis of rat and NMR genes

All the annotated proteins of rat and NMR were searched against Pfam database (containing 13672 families) for the classification into different protein families. 2442 and 2523 protein families (with 2416 overlapped families) were obtained in rat and NMR, respectively, indicating that the two species shared almost the same protein families.

We further analyzed the gain or loss events for each protein family in NMR. Table 1 shows the top 15 families sorted by the number of expanded genes (gain) in NMR. Among them, two distinct cancer-related protein families: "Melanoma-associated antigen family" and "Mortality factor 4 families" had significant gene expansion in NMR. A total of 56 and 15 genes were found for the two families respectively in NMR, which were 27 and 11 more than those in rat. Besides, several other families shown in Table 1 have also been reported to be related to cancer, such as the protein kinase C (PKC) family and several heat shock proteins (HSP70 and HSP90).

**Table 1:** Expanded Pfam family of NMR

PFAM entry	Explanation	class I genes	class II genes	class III genes	expanded
PF00001	rhodopsin family	221	108	205	97
PF01454	Melanoma-associated antigen family	11	18	45	27
PF00433	Protein kinase C family	26	1	27	26
PF02093	Gag P30 core shell protein	1	1	27	26
PF00012	Hsp70 protein	13	0	23	23
PF01849	NAC domain	4	2	20	18
PF00276	Ribosomal protein L23	1	1	16	15
PF01282	Ribosomal protein S24e	2	2	16	14
PF05712	Mortality factor 4 family	2	2	13	11
PF00261	Tropomyosin	5	0	10	10
PF00153	Mitochondrial carrier protein	46	8	17	9
PF00118	TCP-1/cpn60 chaperonin family	12	2	10	8
PF00183	Hsp90 protein	4	0	8	8
PF01015	Ribosomal S3Ae family	1	1	9	8
PF01873	Domain found in IF2B/IF5	2	0	8	8

The Pfam families were sorted by the expanded number of protein families. In this table, we only showed the top 15 families.

The PKC family, which possessed 53 proteins in NMR, had variable roles in tumour biology depending on the intracellular localizations and cell types. PKCs were generally abnormally regulated in the cancers of the breast, prostate, kidney and liver [16], and remained as a possible target for cancer prevention and therapy [17]. Here, a total of 27 additional PKC members were identified in NMR, implying that these new PKCs may play an important role in preventing NMR from cancer.

HSP proteins are a group of functionally related proteins regulating protein folding and unfolding reactions. HSP70 proteins were reported to be overexpressed in the malignant melanoma [18]. On the other hand, HSP90 proteins were also implicated to be involved in breast cancer progression because of its overexpression in breast cancer cell lines and association with survival of breast cancer [19]. Thus, HSP70 and HSP90 proteins have been considered as the useful targets for cancer therapy [20,21]. In this study, the significant expansions of members of these two families in NMR were consistent with their potential roles in cancer prevention and may provide clues for the anti-cancer trait of NMR.

Table 2 shows the top 15 protein families with contracted gene numbers (loss) in NMR. Among them, several protein families have been previously verified to be involved in cancer development. For example, the cadherin family is a class of type-1 trans-membrane proteins. Members of this family may play important roles in cell-cell adhesion in different organ systems [22]. All kinds of abnormal expression of cadherin family proteins had been reported as a widespread phenomenon in mammary cancer and these proteins had been frequently implicated in tumour progression [23]. The loss of so many genes in this family may also contribute to the complex cancer-resistant mechanisms of NMR. In addition, a significant loss of members of some receptor protein families, such as the vomeronasal organ receptor (VOR), olfactory receptor and class C G-protein-coupled receptor, may be important for some unique features of the most unusual rodent.

**Table 2:** Contracted Pfam family of NMR

PFAM entry	Explanation	class I genes	class II genes	class III genes	contracted
PF13853	Olfactory receptor	293	852	691	161
PF03402	Vomeronasal organ receptor	2	99	3	96
PF00003	class C G-protein-coupled receptors	14	72	17	55
PF00028	Cadherin family	50	38	4	34
PF05296	Mammalian taste receptor protein	4	32	2	30
PF08391	Ly49-like protein	1	21	0	21
PF02994	L1 transposable element	0	20	0	20
PF01157	Ribosomal protein L21e	1	32	13	19
PF13885	high sulfur B2 protein	3	12	1	11
PF13841	Beta defensin	7	11	0	11
PF01198	Ribosomal protein L31e	1	16	6	10
PF01779	Ribosomal L29e protein family	1	10	1	9
PF12774	chaperone-like ATPases	1	9	0	9
PF00879	Defensin propeptide	0	9	1	8
PF00618	Ras-like small GTPase	0	8	0	8

The Pfam families were sorted by the contracted number of protein families. In this table, we only showed the top 15 families.

### Pathway analysis of NMR-missing genes

Among 216 rat pathways in the KEGG database, 16 of them were found to be significantly (p-value < 0.01) enriched by the NMR-missing genes (group II genes) (Table 3). For example, at least 31 rat genes (10.7%) in the "Neuroactive ligand-receptor interaction" pathway were not detected in NMR. The loss of these genes might be one of the major reasons why NMR is insensitive to pain and acid [24]. In addition, NMR-missing genes were also significantly enriched in two known cancer pathways, "Bladder cancer" and "Pathways in cancer" (with the p-value 2.37e-3 and 1.17e-3, respectively).

**Table 3:** Pathway enrichment analysis of NMR-missing genes*

pathway ID	pathway name	gene number	total number	p-value
rno04740	Olfactory transduction	518	1015	1.4E-11
rno03010	Ribosome	18	91	4.4E-07
rno04060	Cytokine-cytokine receptor interaction	31	247	2.0E-06
rno04080	Neuroactive ligand-receptor interaction	31	290	5.1E-05
rno00190	Oxidative phosphorylation	26	244	2.1E-04
rno04115	p53 signaling pathway	12	80	5.6E-04
rno05200	Pathways in cancer	29	317	1.2E-03
rno04330	Notch signaling pathway	8	47	2.0E-03
rno05014	Amyotrophic lateral sclerosis	10	69	2.0E-03
rno04610	Complement and coagulation cascades	10	69	2.0E-03
rno05219	Bladder cancer	7	38	2.4E-03
rno04310	Wnt signaling pathway	16	149	3.0E-03
rno04650	Natural killer cell mediated cytotoxicity	14	125	3.6E-03
rno03050	Proteasome	8	52	3.8E-03
rno04210	Apoptosis	12	107	6.8E-03
rno04010	MAPK signaling pathway	19	213	9.9E-03

*: In this table, we only showed the pathways with p-value≤0.01.

Among the rest of pathways shown in Table 3 five of them were thought to be cancer-related, which include "Cytokine-cytokine receptor interaction" [25], "p53 signalling pathway"[26], "Apoptosis", "Natural killer cell mediated cytotoxicity" [27], "Wnt signalling pathway" [28]" and "Notch signalling pathway" [29]. It is possible that several of these NMR-missing genes are associated with cancer development in other mammals including humans and could be considered as candidate cancer-related genes.

As KEGG pathways are composed of nodes which may have single or multiple functionally similar genes, we also calculated the percentage of the nodes which contain at least one NMR-missing gene in each of these pathways, and obtained almost the same enriched pathways, such as "Pathways in cancer", "Neuroactive ligand-receptor interaction" and "Oxidative phosphorylation", implying that the pathway enrichment of NMR-missing genes was significant at both gene and node levels (Supplementary Table 1 [see additional file 1]).

### Analysis of cancer-related genes that were absent in NMR

To investigate the potential mechanisms of the anti-cancer aspects of NMR, three cancer-related pathways, including "pathways in cancer", "MAPK (mitogen-activated protein kinase) signalling pathway" and "Wnt signalling pathway", were chosen as examples for further analysis.

#### 1) Pathways in cancer

Twenty-nine genes in this pathway were not detected in NMR (Supplementary Table 2 [see additional file 1]). These genes were found to be strongly related to cancer. Half of them correspond to various phenotypes of cancer (e.g., leukemia, lung cancer and adrenal cortical carcinoma) based on the information retrieved from OMIM database, including several well-studied carcinogenesis genes (Bcl2, Casp8, Fas). Moreover, some important proto-oncogenes, such as Myc and Hras1, were also absent. In fact, it is well known that proto-oncogenes are normal genes that could become the oncogenes because of their overexpression or mutations. The loss of the proto-oncogenes in NMR cells may also contribute to cancer resistance.

Among all 29 NMR-missing genes in this pathway, 19 of them (65.5%) were previously reported to display differential gene expression levels between cancer and normal tissues. Thus, the absence of these genes might play important roles in suppressing cancer.

**Figure 2:**
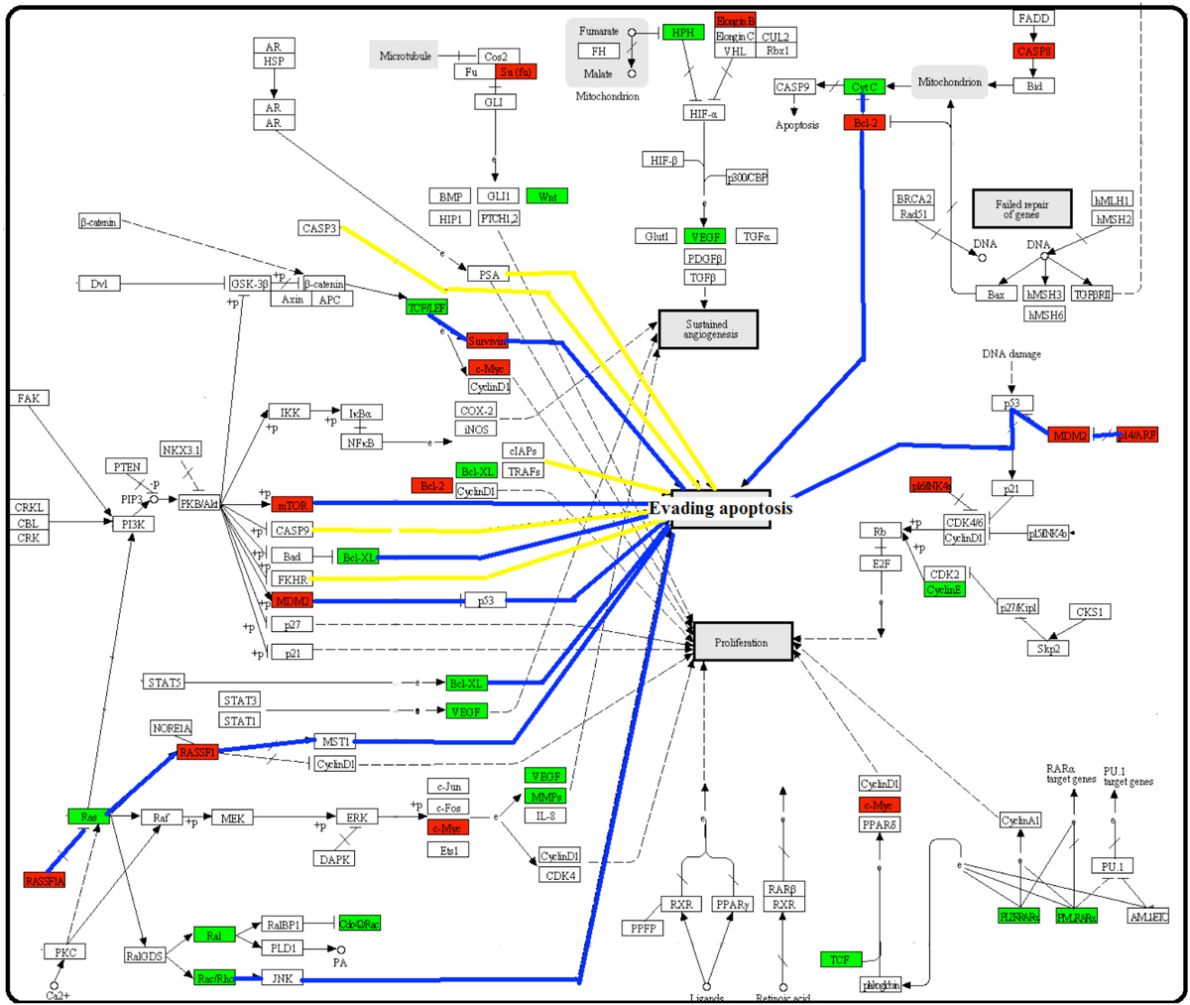
**pathways in cancer**. Green nodes represent the nodes in which parts of genes were lost, whereas red nodes represent the nodes in which all genes were lost. White nodes represent the nodes which were same between rat and NMR. A blue line denotes the path of evading apoptosis containing at least one red or green node, whereas a yellow line denotes the same path between rat and NMR. In this figure, nine of fourteen paths leading to evading apoptosis have been affected.

Figure 2 shows the main part of the map of "pathways in cancer". One of the key mechanisms for cancer development is "Evading apoptosis", which is a crucial oncogenic property of cancer cells. Among the 14 paths leading to evasion of apoptosis illustrated in this map, 9 of them appeared to be influenced by direct connection to the nodes which contain NMR-missing genes. Particularly, the nodes of Survivin, Bcl-2, Bcl-XL and Mtor have lost several functionally similar genes in NMR. It is possible that these changes in NMR might block the major routes to evade apoptosis, and then induce the early programmed death of cancer cells as an important cancer barrier.

Recently, a two-tier anti-cancer mechanism associated with contact inhibition regulated by p16^Ink4a ^and p27^Kip1 ^has been reported in NMR [30]. However, rat cells were found to only have contact inhibition regulated by p27^Kip1^. This is consistent with our results as rats only have p27^Kip1 ^gene. Thus, NMR appeared to have additional unique protective mechanisms for cancer resistance.

#### 2) MAPK signalling pathway

The MAPK signalling pathway is an important pathway how proteins in the cytoplasm communicate the signals from the receptors on the cell membrane to the nucleus. It is in the central of a molecular metabolic network that mediates cell differentiation and proliferation. In mammalian cells, the MAPK pathway contains three major groups of proteins, including Erk (extracellular signal-regulated kinase), p38 kinase and Sapk (stress activated protein kinase). These proteins are abnormally regulated in various diseases, including cancer and inflammation.

**Figure 3:**
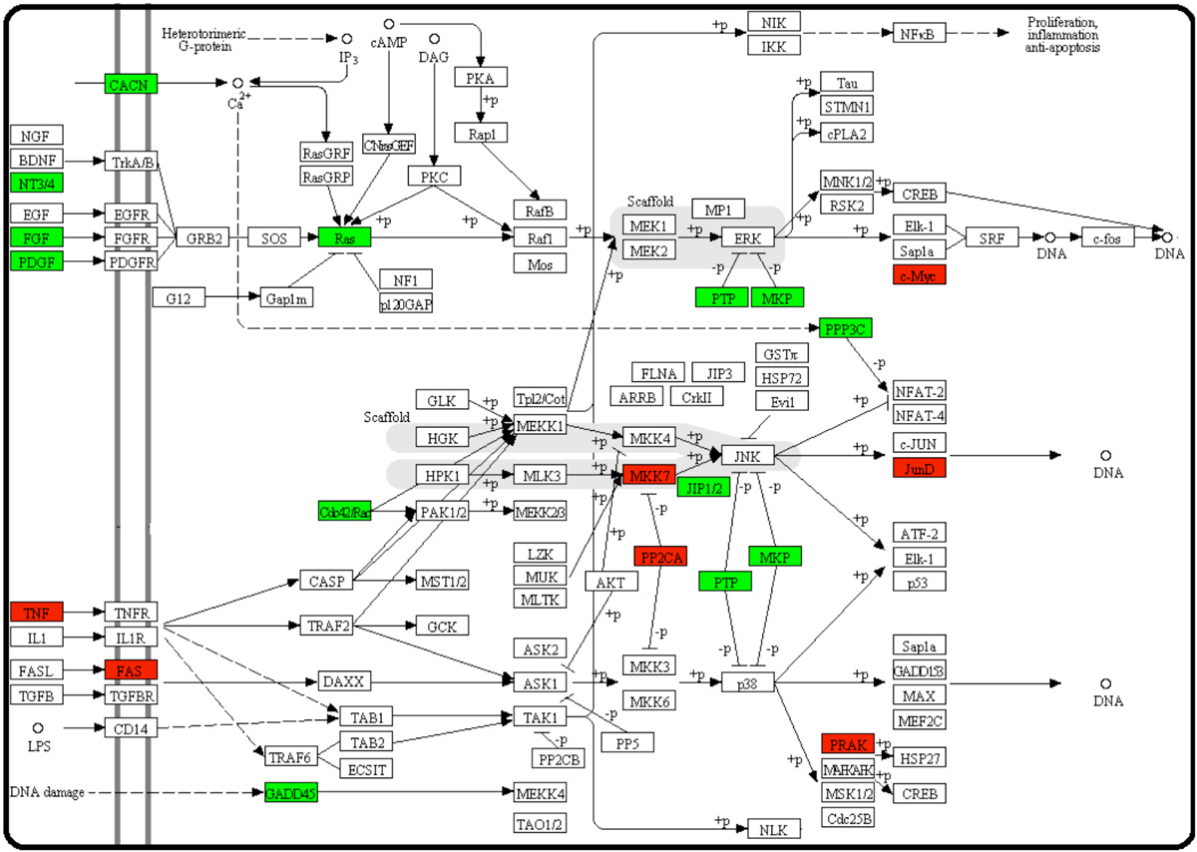
**MAPK signalling pathway**. See Figure 2 legend for the definitions of different colours. In this pathway, many important cancer-related genes, such as Hras1, Mkk7 and Prak, were lost.

In this study, 19 genes were found to be absent in NMR (Figure 3 and Supplementary Table 3 [see additional file 1]). By retrieving the OMIM database and GXA database, we found that 7 (36.8%) of them have been reported to be related to various phenotypes of cancer in other mammals and 10 (52.6%) of them had differential expression levels between cancer and normal tissues.

In this pathway, three proto-oncogenes, Hras1, Myc and Pdgfb (also present in the "Pathways in cancer" pathway), were absent in NMR. It has been previously shown that when one of these genes was mutated, the activity of their enzymes could be stuck in the "on" or "off" position, which was an essential step during the development of many cancers [31]. Recently, it has also been reported that NMR and rat cells acted totally opposed if transfected with Hras1. NMR cell cycle came to an abrupt end as the presence of abnormal chromatin material and anaphase bridges and, while transfected rat cell grew rapidly and formed tumours eventually [32]. Therefore, the loss of these genes might also play a significant role in cancer resistance.

Although the phenotypes of cancer could not be found for other NMR-missing genes, some of them have been demonstrated to be related to the survival of cancer cells, such as Jund and Park genes. Jund is an AP-1 family member involved in various biological processes such as cell apoptosis and tumour metastasis, and could regulate survival of tumour cells in prostate cancer [33]. Prak is a protein kinase, which was previously shown to be implicated in the suppression of skin carcinogenesis [34]. Further experiments are needed to investigate the relationship between these genes and tumorigenesis.

#### 3) Wnt signalling pathway

The "Wnt signalling pathway" is a conserved protein-protein interaction network that regulates cell fate decisions and cell-cell communication. This pathway plays a significant role in maintaining stability of internal environment by regulating cell niche *in vivo*. Abnormal regulation of this pathway could lead to neoplastic proliferation which is involved in the progress of cancer cells.

**Figure 4:**
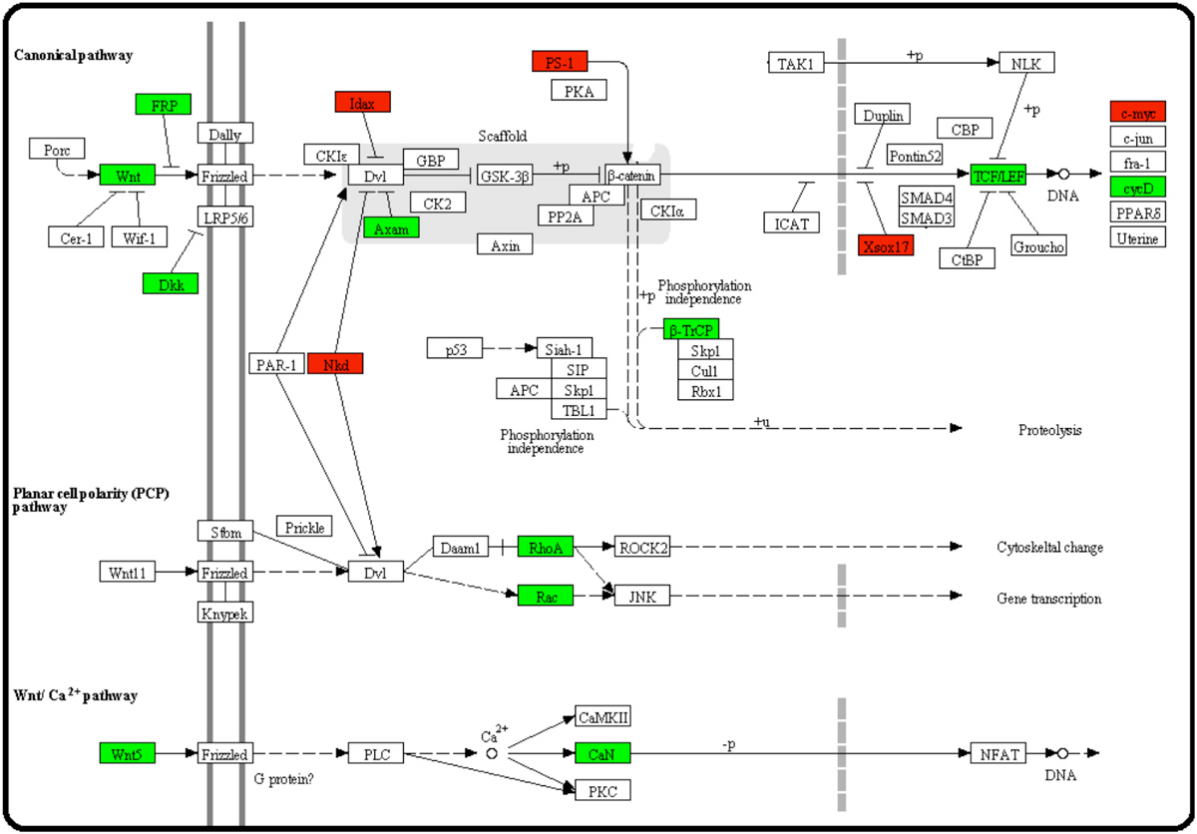
**Wnt signalling pathway**. See Figure 2 legend for the definitions of different colours. Some well-studied cancer-related genes such as Lef1, Rhoa and Rac1, were lost in this pathway.

Seventeen genes were found to be absent in the NMR genome (Figure 4 and Supplementary Table 4 [see additional file 1]). Among them, 4 (25%) genes have corresponding phenotypes of cancer whereas 12 (75%) genes were differentially expressed between cancer and normal tissues. Molecular-level studies have indicated that approximately 90% of the activating mutations of genes in this pathway could cause different cancers such as colorectal cancer [35].

Several well-studied cancer-related genes, such as Myc, Rhoa, Lef1 and Rac1, were absent in this pathway of NMR. Myc is a well-known proto-oncogene and has been frequently used to induce tumour formation in a lot of animal experiments of cancer research. Rhoa has been deeply studied and proved as a cancer-regulated gene, which controlled metastasis of tumour cells, acted as a regulator of male hormone activity in prostate cancer cells [36], and triggered a particular microvesicle signalling pathway in cancer cells [37]. Lef1 protein could interact with a number of other proteins, such as Ctbp and Nlk. These interactions were thought to be responsible for the invasion and growth of prostate cancer [38]. Rac1 was found to be associated with DNA transcription. Previous studies have reported that activation of Rac1 mediated Twist1-induced cancer cell migration [39]. On the other hand, 12 of the NMR-missing genes in this pathway, such as Dkk4, Sox17 and Ccnd3, have not been reported to be related to any disease including cancer. It is possible that some of them are also involved in cancer formation and could be further experimentally verified.

### Sequence variation analysis of Class I genes

Based on the BLASTP sequence alignment results, we found that 6349 (41.2%) of the orthologs had no gaps and 12142 (78.8%) orthologs only possessed a small (≤5 amino acids) gaps, suggesting that most of the orthologous proteins between rat and NMR had rare insertions/deletions during evolution. On the other hand, 439 orthologous proteins showed significant segment insertions/deletions whose length was more than 25 amino acids. Further analysis of these inserted and deleted fragments in these proteins revealed that many of them contained conserved sites, including functionally active sites (Supplementary Table 5 [see additional file 1]). For example, we observed that parts of the specific RNA/DNA binding site and the specific cytokine receptor motif were deleted in the NMR Fusip1 and Nrcam proteins, respectively.

Other domains affected by the insertion/deletion of certain segments included ATP-binding, Ca^2+ ^binding and some other metal catalytic binding sites. For example, a 30-amino-acid-long sequence fragment was found to be inserted into the putative catalytic site in NMR Ship1 when compared with its ortholog in rat. It has been previously demonstrated that the phosphate domain of Ship1 was essential for catalytic activity in vivo [40] and the loss of Ship1 could promote leukemogenesis in a virus-infected mouse model [41]. We suspect the insertion of such a long segment of Ship1 would change the function or expression of this gene. On the other hand, wrong annotation of some NMR proteins could not be excluded. Further studies are required to verify the presence of the sequence variations and their influence on the regulation or function of these proteins.

## Conclusions

In this paper, a comparative genomics study was carried out to investigate the genes that were either common between rat and NMR, or specific to each of them. The majority of genes were shared by the two rodents, whereas each organism had a significant part of unique genes. Seven cancer-related protein families, such as melanoma-associated antigen family, protein kinase C family and HSP family were found to be significantly expanded. Further analysis of the genes absent in NMR indicated that the majority of them have been shown to be linked to many forms of cancer. Finally, some conserved functional domains were found to be possibly influenced by the insertion or deletion of certain fragments in NMR, which may change the expression or function of some of these genes. These results may provide important clues about the molecular mechanisms of cancer resistance of NMR and help identify new cancer-related genes [42] in mammals. As future topics, it is important to study such complex mechanisms from the viewpoints of network [43-46] and dynamics [47,48] by further incorporating the expression data.

## List of abbreviations

NMR: naked mole rat; KEGG: Kyoto Encyclopaedia of Genes and Genomes; NCBI: National Center for Biotechnology Information; GXA: Gene Expression Atlas; CDD: Conserved Domain Database; OMIM: Online Mendelian Inheritance in Man; PKC: protein kinase C; HSP: heat shock proteins; TERT: telomerase reverse transcriptase; GPx1: glutathione peroxidase 1; VOR: vomeronasal organ receptor

## Competing interests

The authors declare that they have no competing interests.

## Authors' contributions

YZ and LC designed the study. ZY carried out computational studies, including comparative genomics, sequence alignment, Pfam and KEGG analysis and wrote the manuscript. YZ and LC analyzed the data and edited the manuscript. All authors read and approved the final manuscript.

## Supplementary Material

Additional file 1 **The table contains supplementary table 1-5**. Supplementary table 1: The percentage of nodes containing NMR-missing genes in each pathway. Supplementary table 2: NMR missing genes in "Pathways in cancer". Supplementary table 3: NMR missing genes in "MAPK signalling pathway". Supplementary table 4: NMR missing genes in "Wnt signalling pathway". Supplementary table 5: Sequence variation of Class I genes. Click here for file

## Declarations

The publication of this article is funded by grants from the National Natural Science Foundation of China (91029301, 61134013, 31171233, 61072149), a grant from Chinese Academy of Sciences (CAS) (2012OHTP10), the Chief Scientist Program of Shanghai Institutes for Biological Sciences of CAS (2009CSP002), and the FIRST program from JSPS initiated by CSTP.

This article has been published as part of *BMC Systems Biology *Volume 7 Supplement 2, 2013: Selected articles from The 6th International Conference of Computational Biology. The full contents of the supplement are available online at http://www.biomedcentral.com/bmcsystbiol/supplements/7/S2.
